# Deep-sea megabenthos communities of the Eurasian Central Arctic are influenced by ice-cover and sea-ice algal falls

**DOI:** 10.1371/journal.pone.0211009

**Published:** 2019-07-16

**Authors:** Elena Rybakova, Antonina Kremenetskaia, Andrey Vedenin, Antje Boetius, Andrey Gebruk

**Affiliations:** 1 Shirshov Institute of Oceanology, Russian Academy of Sciences, Moscow, Russia; 2 Alfred Wegener Institute, Helmholtz Centre for Polar and Marine Research, Bremerhaven, Germany; 3 Max Planck Institute for Marine Microbiology, Bremen, Germany; 4 MARUM, University of Bremen, Bremen, Germany; Museum National d'Histoire Naturelle, FRANCE

## Abstract

Quantitative camera surveys of benthic megafauna were carried out during the expedition ARK-XXVII/3 to the Eastern Central Arctic Basins with the research icebreaker *Polarstern* in summer 2012 (2 August-29 September). Nine transects were performed for the first time in deep-sea areas previously fully covered by ice, four of them in the Nansen Basin (3571-4066m) and five in the Amundsen Basin (4041-4384m). At seven of these stations benthic Agassiz trawls were taken near the camera tracks for species identification. Observed Arctic deep-sea megafauna was largely endemic. Several taxa showed a substantially greater depth or geographical range than previously assumed. Variations in the composition and structure of megabenthic communities were analysed and linked to several environmental variables, including state of the sea ice and phytodetritus supply to the seafloor. Three different types of communities were identified based on species dominating the biomass. Among these species were the actiniarian *Bathyphellia margaritacea* and the holothurians *Elpidia heckeri* and *Kolga hyalina*. Variations in megafaunal abundance were first of all related to the proximity to the marginal ice zone. Stations located closer to this zone were characterized by relatively high densities and biomass of *B*. *margaritacea*. Food supply was higher at these stations, as suggested by enhanced concentrations of pigments, organic carbon, bacterial cell abundances and nutrients in the sediments. Fully ice-covered stations closer to the North Pole and partially under multi-year ice were characterized by lower concentrations of the same biogeochemical indicators for food supply. These stations nevertheless hosted relatively high density and biomass of the holothurians *E*. *heckeri* or *K*. *hyalina*, which were observed to feed on large food falls of the sea-ice colonial diatom *Melosira arctica*. The link between the community structure of megafauna and the extent and condition of the Central Arctic sea-ice cover suggests that future climate changes may substantially affect deep ocean biodiversity.

## Introduction

Benthic megafauna comprises marine animals exceeding 0.5–1 cm in size visible on seafloor images. They play an important role in benthic ecosystems through active recycling of sedimented organic matter, bioturbation and food web linkages. Megabenthos is a dynamic component of deep-sea ecosystems able to react rapidly to environmental changes [1]. Observations of benthic megafauna in the Central Arctic Basins are rare because of technical difficulties of sampling in the remote deep-sea region covered by permanent ice. Traditional sampling methods such as trawling are challenged by the ice-cover: vessels cannot keep steady speed and course in the ice. Hence, most previous studies of Arctic megafauna communities were confined to marginal seas [2, 3, 4, 5, 6, 7, 8, 9, 10, 11, 12, 13], the Fram Strait and areas around Svalbard [14, 15, 16, 17, 18, 19, 20]. Sampling of Central Arctic benthic megafauna from depths exceeding 2000 m began in the late 19^th^ century [21, 22, 23, 24, 25, 26, 27, 28, 29]. Further contributions were made by Soviet expeditions [30, 31, 32] and by the drifting ice stations [33, 34, 32]. Quantitative studies of the Central Arctic megafauna are few and they are focused on the Canada Basin [35, 36, 37].

First extensive photographic observation of Arctic deep-sea megafauna was conducted in the second half of the 20^th^ century [38]. More recent photographic and video surveys of Arctic deep-sea megafauna were conducted in the Canada Basin in 2002 [39] and 2005 [40]. Authors of those studies considered most data as qualitative. So far, the only knowledge of temporal and spatial variation of Arctic deep-sea megafauna based on photo transects was confined to the HAUSGARTEN observatory in the Fram Strait, between Spitzbergen and Greenland [14] at depths of 1200-5500m.

Recent compilations of data on Arctic benthos confirm a strong decline in abundance and biomass with depth from the outer shelves to the inner basins of the Arctic Ocean [13, 41]. A remarkable characteristic of the Central Arctic deep-sea megafauna is its very low density, as previously shown for the Canada Basin at depths of 3816–3843 m [40]. Similar patterns were described for the Arctic deep-sea macrofauna [42]. In the Arctic Ocean abundance and biomass also depend on ice cover [14]. Kröncke [42, 43] and Kröncke et al. [44] suggested that the ice-covered Arctic Eurasian Basin is one of the most oligotrophic regions of the world ocean. In a more recent investigation, Degen et al. [45] combined data from modern field studies with published and unpublished data from the past 20 years and confirmed that the abundance, biomass and production of benthic macrofauna were the lowest under the full, multi-year ice-cover, but increased close to the productive marginal ice zone. The recent synthesis of Vedenin et al. [46] for macrofauna also showed substantial relationships of quantitative characteristics with water depth and sea ice cover that affect the food input to the deep sea. Energy flux via deposition of photosynthesis based primary produced organic matter is the key factor determining the abundance and biomass of benthic communities in the deep Arctic Ocean [47, 48, 49, 50, 51, 52, 53].

Other important characteristics of communities are diversity and community composition. Those characteristics are controlled by such factors as availability of the hard substrate, currents, proximity to shelves, geophysical properties and evolutionary history of basins [49, 54, 55]. According to Mironov et al. [56] the biogeographical history of the Arctic Ocean is characterized by processes of fauna emergence and submergence.

During the past four decades, significant reductions of the sea-ice cover and thickness and extension of the melting season were observed in the Arctic, and they are expected to continue as a result of global climate change [57, 58, 59, 60, 61, 62, 63]. Sea-ice retreat leads to increasing light availability and this in turn may increase photosynthesis based primary production [64, 65]. On the other hand ice melt lead to increasing of ocean stratification and accordingly to decreasing nutrient availability and this in turn may decrease photosynthesis based primary production [66, 67, 68]. Average estimates of photosynthesis based primary production in the ice-covered Central Arctic are low [69, 66]. Ice algae (algae attached to the bottom surface of the ice or living in the ice itself) can be a key food source in Arctic marine food webs [70, 71]. Hence sea-ice loss could lead to decreased ice algal production and sedimentation of ice algae on the sea floor affecting the quantity and quality of food available to benthic communities. Deposition to the seafloor of large aggregations of the sea-ice diatom *Melosira* is known for Arctic shelves [72, 73] and was observed to occur in the deep, ice-covered basins as a consequence of rapid sea-ice melt in 2012 [71]. However the overall contribution of the ice diatoms to the nutrition of benthos remains unknown, only limited data exists for shelf and slope communities of the Chukchi, Barents and Bering Seas [74, 72, 73].

This study on the ice-covered Nansen (3571–4066 m) and Amundsen (4041–4384 m) Basins was conducted in the summer of 2012, during the record minimum of sea ice cover in the Arctic Ocean [71]. Using the towed video and photographic platform Ocean Floor Observation System (OFOS), massive accumulations of live and degraded ice diatom algae were observed for the first time in the basins of the Arctic Ocean at the depth of ~ 4000 m [71]. It was recorded that some benthic animals, foremost holothurians and ophiuroids, actively feed on algal patches, and this was confirmed by the study of gut contents of these species [71].

In the present study we investigated the key factors structuring the distribution of abyssal megafauna in the Central Arctic, including variations in sea-ice cover and biogeochemical variables indicating food supply by phytodetritus deposition. Our main aims included revealing 1) the structure of megafauna communities in the ice-covered basins and effect of the marginal ice zone and 2) the effect of algal food falls on megafauna. Also, we compared the Eurasian basin megafauna community composition and structure with that of adjacent regions, and identified several new depth and geographical records for a number of taxa. Obtained data provide a baseline for future studies in the changing deep-sea Arctic Ocean.

## Material and methods

### Study area, photographic survey and sampling

**Fig 1:**
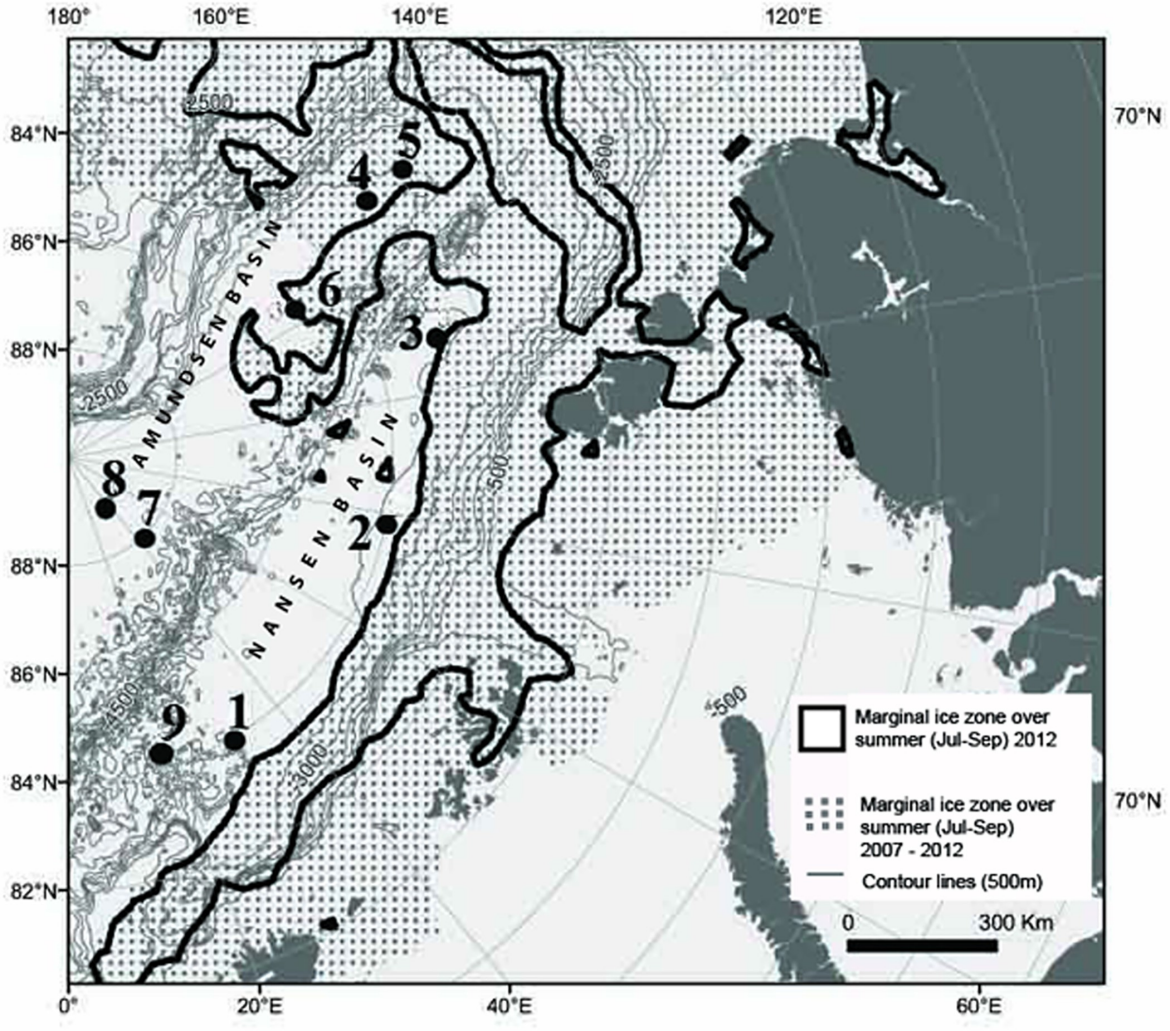
Location of stations performed during the expedition ARK-XXVII/3 in summer 2012 (August-September) to the Arctic Ocean. Ice margin in 2012 (black line) and the marginal ice zone integrated over previous 5 years (dotted area) where sea ice data were available are shown. 2012 represented a new sea-ice minimum except for the area around Gakkel Ridge (140–180°E) that showed a minimum already in 2007. Stations 1–5 were situated closer to the marginal ice zone and were characterized by the first-year sea ice. Stations 7–9 were situated at some distance from the ice edge and/or were characterized by multi-year ice (6).

Photographic surveys were carried out during the expedition ARK-XXVII/3 in summer 2012 (2 August-29 September) in the Nansen and Amundsen Basins for the first time in deep-sea areas previously fully covered by ice (Fig 1). Seafloor was photographed using a towed Ocean Floor Observation System (OFOS) [75]. Nine stations separated from each other by 52–689 nautical miles distance (one transect per station) were performed: four in the Nansen Basin (1,2,3,9) between 83–84°N and 18–110°E at depths 3571–4066 m, and five in the Amundsen Basin (4,5,6,7,8) between 83–89°N and 56–131°E at depths 4041–4384 m (Fig 1). Stations 1–5 were situated closer to the marginal ice zone (MIZ) and were characterized by first-year sea ice (FYI), whereas stations 7–9 were situated at some distance from the ice edge and were characterized by multiyear ice (MYI) [71]. Station 6 was somewhat closer to the ice edge in 2012 than in any previous year. Total analysed area of the seafloor comprised 16190 m^2^, ranging from 206 m^2^ to 3379 m^2^ per transect. Length of transects varied from 210 m to 5500 m (Table 1). Marginal ice zone was defined as the zone showing 30–50% of the ice cover.

**Table 1:** Details of OFOS transects and trawl sampling (station number, location, date, coordinates, survey time, water depth, area covered by OFOS images and estimated trawled area, area covered by OFOS images used in statistical analysis, number of OFOS images and number of OFOS images used in statistical analysis, OFOS transect length).

Station number	Location	Deployment	Date, 2012 (dd.m)	Position, North / East (°)				Survey time (min)	Water depth (m)		Area covered by OFOS images/estimated trawled area (m^2^)	Area covered by OFOS images used in statistical analysis (m^2^)	Number of OFOS images	Number of OFOS images used in statistical analysis	OFOS transect length (M)
				Start		End			Start	End					
115501	Nansen Basin	OFOS PS80_228–1	10.8	84.0085	31.3647	83.9979	31.4261	258	4010	4011	2361	2308	643	637	1550
		Trawl PS80_222–1	09.08	84.0377	30.1620	84.0380	30.1878	47	4012	4013	1050				
2	Nansen Basin	OFOS PS80_239–1	14.8	83.9780	78.0070	83.9686	77.5569	315	3477	3468	3721	3379	958	941	5500
		Trawl PS80_249–2	17.08	83.9720	77.6825	83.9707	77.6315	15	3470	3470	2353				
3	Nansen Basin, close to Gakkel Ridge	OFOS PS80_257–1	20.8	82.6860	109.5978	82.727733	109.6061	236	3572	3575	2989	2085	719	675	4380
		Trawl PS80_259–1	20.8	82.7090	109.5777	82.7235	109.5625	55	3575	3576	4800				
4	Amundsen Basin near Gakkel Ridge	OFOS PS80_282–1	26.8	82.8939	129.8822	82.8796	129.8585	270	4168	4172	2739	2259	790	739	1620
		Trawl PS80_286–1	26.8	82.7905	129.8777	82.7780	129.8467	73	4158	4159	4789				
5	Amundsen Basin	OFOS PS80_327–1	4.9	81.9178	130.925	81.8894	130.8894	250	4042	4033	2170	1871	745	731	3150
		Trawl PS80_332–1	5.9	81.9093	130.8767	81.9062	130.8432	18	4038	4039	2421				
6	Amundsen Basin	OFOS PS80_340–1	8.9	85.0587	122.6933	85.0854	122.8348	472	4351	4354	3668	3122	1289	1131	3700
		Trawl PS80_346–1	9.9	85.0725	122.7070	85.0688	122.6915	15	4353	4354	1461				
7	Amundsen Basin	OFOS PS80_356–1	19.9	87.9260	61.1264	87.9240	61.1493	180	4384	4383	223	206	428	81	260
		Trawl PS80_359–1	19.9	87.8922	59.3887	87.8962	59.3468	15	4380	4380	1400				
8	Amundsen Basin	OFOS PS80_369–1	23.9	88.7807	56.1396	88.7749	56.0243	157	4375	4375	698	671	466	226	750
9	Nansen Basin, close to the Gakkel Ridge	OFOS PS80_392–1	29.9	84.3533	17.7070	84.3516	17.7136	74	4049	4067	296	289	225	111	210
**Total**								**2212/238**			**18865/18274**	**16190**	**6263**	**5272**	**21120**

The OFOS platform was equipped with the Canon camera EOS-1Ds Mark III (modified for underwater applications by iSiTEC GmbH, Germany), the strobe Kongsberg 0E11-242, four LED lights (LED Multi-Sealite, DeepSea Power and Light), telemetry (LRT-400 Fiber, iSiTEC) and three red laser points (OKTOPUS). A triangle laser scale with 50 cm between lasers was used to determine the camera’s footprint. Still camera was mounted to the frame in a vertical position to the seafloor. It was triggered automatically every 20 s, resulting in about 10–110 images per 100 m (for more details see Meyer et al. [75] and Soltwedel et al. [14]). OFOS was towed at approximately 0.1–1 knots in a ship drift for 1.5–8 hour of bottom time at a target altitude of 1.3 m above the seafloor. This altitude has proven to be the optimal distance to the seafloor to achieve the best illumination and resolution of the images [14]. The area of the seafloor on each image varied from 1 to 9 m^2^ depending on altitude, in most cases it was 3–4 m^2^. Start and end positions of transects (from GPS fixes) and water depths along transects (from echo soundings) were obtained from the ship’s data acquisition and management system DSHIP. Transect details, such as location, duration and the number of images are given in Table 1. The entire dataset can be downloaded from doi.pangaea.de/10.1594/PANGAEA.896626.

Agassiz trawl samples (frame width 3m, mesh size 2cm) were taken in the immediate vicinity of the photographic transects to obtain specimens for verification of taxonomic identifications based on images (Table 1). Therefore, the ship was returned to the start coordinates of the OFOS transect and then followed approximately the same course as in the OFOS deployment. No specific permissions were required for all investigated locations because they are not the protected areas. In areas of stations 8 and 9 the thick ice prevented trawling operations. At stations 3, 4 and 7 the total trawled area (per station) was >1.5 times larger than the photographed area of the seafloor due to very low drift speed (<0.5 kn). At other stations the total trawled area was almost the same or two times smaller than the photographed area. Trawl samples were washed through a sieve (1 mm mesh size), sorted and preserved in 4% buffered formaldehyde; specimens with calcareous skeleton were preserved in 80% ethanol. Afterwards all preserved specimens were counted and identified in the lab. The field studies did not involve endangered or protected species.

### Image analysis

All images were analysed and stored using the image analysis program and database BIIGLE (Bio-Image Indexing, Graphic Labelling, and Exploration) web-2.0, which can be accessed by any standard web browser (www.BIIGLE.de) [15, 76, 77]. Laser points were automatically enhanced by BIIGLE software, and used for calculation of the seafloor surface area on images in an automated way using a BIIGLE subroutine [77]. At transects 1–6 there was no overlap between images and each image was treated as a separate sample. At transects 7–9 some overlap between images occurred owing to the low drift speed, however images partly overlapping were excluded from the analysis. Remaining images were also treated as separate samples. Images of unsatisfactory quality (with sediment clouds, too strong or low illumination, deviating distance from the bottom) were also excluded from the analysis. In total 6263 digital images were examined. Of these, 5272 images that met selection criteria were used for statistical analyses (Table 1).

All taxa were labelled in BIIGLE by species/feature name selected from a drop-down list [78]. Visible megafauna was counted and identified to the lowest possible taxonomic level. Taxonomic identifications were made with the assistance of zoological experts (see acknowledgments). For identifications of some species, specimens from trawl catches were used, others were identified only based on images. The following taxa/organisms were excluded from statistical analyses because they could not be adequately identified and counted on images: infauna represented only by Lebensspuren, gelatinous zooplankton, small-size organisms (< 1 cm) and organisms that could not be identified at least to the phylum level. Complete list of taxa identified on transects and in trawl samples can be downloaded from doi.pangaea.de/10.1594/PANGAEA.896618.

Coverage of seafloor by algal aggregations and their remains was calculated based on manual analysis of 60 images at each transect using ImageJ software [79]. Images for this analysis were chosen with equal spatial intervals. Degree of freshness of algae in aggregations was evaluated visually using the two categories: greenish-brownish as freshly deposited, and whitish-yellowish as mostly degraded diatom falls.

### Environmental parameters

Several environmental parameters were measured at stations to assess possible effect on megabenthos communities. In the top 0–1 cm of sediment we analysed concentrations of chlorophyll a [Chl a], porosity (%), abundances of bacterial cells, total organic carbon (TOC), dissolved organic carbon (DOC), total dissolved nitrogen (TDN) and concentration of nutrients (PO_4_, Si, NO_2_, NO_3_). Those parameters were measured in samples taken using a TV-guided multicorer (two samples per station). Multicorers were targeted to the same area as OFOS transects by GPS positioning. For more details of sediment characteristics measurements see Rossel et al. [53, 80]. All data from ARK-XXVII/3 were submitted to the Earth system database PANGAEA (see reference list for data collections [81, 82, 83, 84, 85, 86]). Characteristics of the sea ice included thickness, age and percentage of the ice cover. They were measured using airborne electromagnetic induction sounding during the helicopter surveys [87].

### Data analysis

Trawl data was used only for qualitative taxonomical studies to supplement species list obtained based on images. We did not use trawl data for quantitative analysis. There are difficulties with the estimation of the seafloor area sampled by trawl, because the exact bottom contact time is not known. Also trawl sampling is inefficient in catching mobile epibenthic organisms because they escape trawls. When caught in the trawl, organisms are often destroyed by massive amount of sediment, or washed through the net when they are small. On the other hand, the trawl collects infauna that is not visible on images. Number of taxa was calculated at each station separately based on images and trawl samples, and together based on images and trawls.

Quantitative analysis was performed only for image data. Mean taxa densities (±standard deviation) and total megafauna density (±standard deviation) were calculated for each transect. Densities of each taxa were calculated per image and then average density per transect was obtained. Relative contributions (%) of the most abundant taxa to the total density were estimated. Following diversity indices were applied to describe megafauna assemblages: Pielou’s evenness (J'), Shannon–Wiener diversity (H') (log _2_) and Simpson’s diversity (1-λ).

Density data based on images were square-root transformed to reduce the dominance of the most abundant species. Bray-Curtis similarity coefficient was calculated using square-root transformed data. Non-metric multidimensional scaling plot (NMDS) was generated. Contribution of taxa to similarity and dissimilarity of different groups of stations was calculated using the SIMPER subroutine of PRIMER v6 [88].

Mean taxa biomass per m^2^ and the total megafauna biomass per m^2^ were estimated for each transect. Mean biomass (preserved wet weight) was calculated based on the wet weight of preserved individuals sampled by trawls. Average wet weight for the entire taxon identified to the lowest possible taxonomical level in a trawl was calculated and applied to all individuals of that taxon on image transects, regardless of visual size. For taxa with insufficient trawl data the biomass was estimated using the biomass data of taxonomically similar taxa or taxa with similar body shape.

Percentage of holothurians *Kolga hyalina* and *Elpidia heckeri* and the ophiuroid *Ophiostriatus striatus* associated with fresh and detrital algae aggregations was calculated for each transect in order to assess the contribution of algae to the nutrition of those species.

Spearman's rank correlation was calculated between the environmental/sediment parameters and community characteristics or taxa densities/biomass. A canonical correspondence analysis (CCA) was performed at the station scale to estimate the contribution of environmental and sediment parameters to megafauna structure (DOC and TDN were not included because they were not measured at stations 1 and 5) [89] based on images. Statistical analyses were performed in Primer V6 [90] and PAST V3 [91].

Biogeographic distribution patterns were evaluated for 43 species and 55 genera (all reliable identifications based on images and trawl samples).

## Results

### Imagery analysis

#### Taxonomic composition

In total 5272 images were analysed corresponding to the total area of 16190 m^2^ of the sea floor (Table 1). Overall 55000 specimens from 58 taxa were recognised, of which 51 taxa occurred in the Amundsen and 47 taxa in the Nansen Basin. Seven taxa were unique for the Nansen Basin and 11 for Amundsen Basin. Number of taxa per transect varied from 19 (St. 7) to 43 (St. 6).

**Fig 2:**
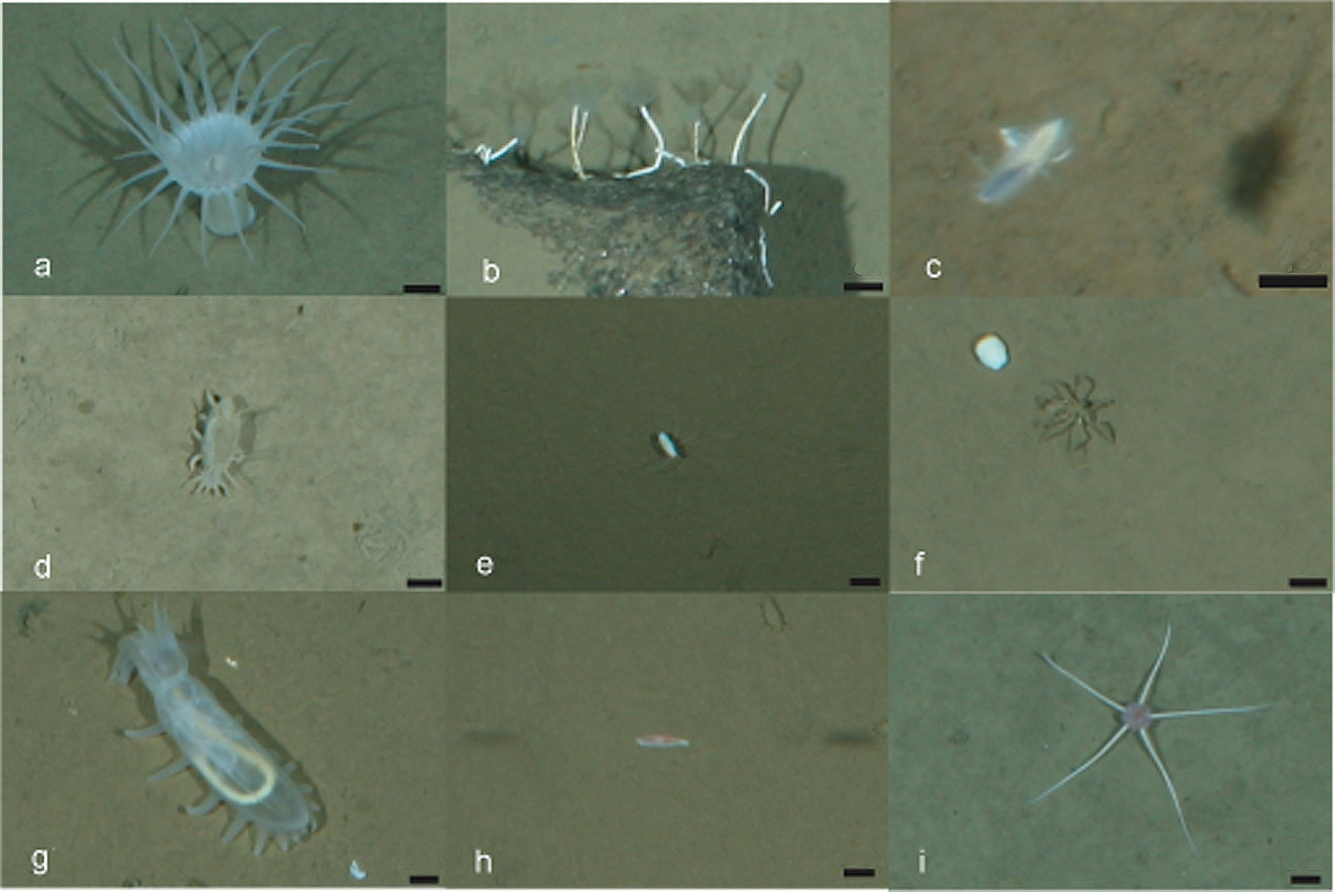
Images of the most abundant taxa. (a) *Bathyphellia margaritace* (Actiniaria), (b) *Hyalopomatus claparedii* (Polychaeta), (c) Macellicephalinae gen.sp.5 (Polychaeta), (d) *Elpidia heckeri* (Holothuroidea), (e) *Eurycope inermis* (Isopoda), (f) *Ascorhynchus abyssi* (Pycnogonida), (g) *Kolga hyalina* (Holothuroidea), (h) *Onisimus leucopis* (Amphipoda), (i) *Ophiostriatus striatus* (Ophiuroidea). Scale bar 1 cm.

Six following taxa were abundant and common at all transects: the actiniarian *Bathyphellia margaritacea*, the serpulid polychaete *Hyalopomatus claparedii*, the polychaete Macellicephalin gen. sp.5, the isopod *Eurycope inermis* and the holothurians *Elpidia heckeri* and *Kolga hyalina* (Fig 2). Eleven taxa were also common but less abundant, i.e. present on ~25% of images: the sponge Porifera gen. sp., the actiniarian Actiniaria gen. sp.1, unidentified animals in holes, the ceriantharian *Cerianthus* sp., the polychaete Macellicephalin gen. sp.1, the gastropod *Mohnia* sp., the amphipod *Eurythenes gryllus*, the lysianassid amphipod *Onisimus leucopis*, the mysid Mysidae gen. sp., the pycnogonid *Ascorhynchus abyssi* and the chaetognath *Pseudosagitta maxima*. Twelve taxa were referred to “rare” as they were observed only on one of the transects on maximally 5 images (doi.pangaea.de/10.1594/PANGAEA.896618).

Thirty taxa were identified to the species or genus level, 16 to family; others were assigned to higher taxa. Number of taxa belonging to major taxonomical groups are presented in Table 2. The most species-rich group was the cnidarians with 13 taxa. The majority of polychaetes, with at least eight taxa identified to the subfamily level, belonged to the Macellicephalinae. The amphipods of the family Lysianassoidea with five taxa (most of them at the family level) were the most diverse.

**Table 2:** Number of taxa in major taxonomic groups (image data, trawl data, images and trawl data, total).

Taxon	Images	Trawl samples	Images and trawl data	Total
Porifera	4	7	3	8	
Cnidaria	13	7	3	17	
Polychaeta	11	10	2	19	
Nemertea	2	1	1	2	
Bivalvia	1	4	1	4	
Gastropoda	3	2	0	5	
Cephalopoda	0	1	0	1	
Isopoda	3	1	1	3	
Amphipoda	11	7	2	16	
Mysida	1	0	0	1	
Decapoda	1	3	1	3	
Pycnogonida	1	1	1	1	
Bryozoa	1	2	1	2	
Ophiuroidea	1	1	1	1	
Crinoidea	1	1	1	1	
Holothuroidea	2	2	2	2	
Asteroidea	0	1	0	1	
Chaetognatha	1	0	0	1	
Enteropneusta	1	0	0	1	
**SUM**	**58**	**51**	**20**	**89**	

### Community structure

Taxa richness was the highest at the ice-covered station 6 in the vicinity of Gakkel Ridge and the lowest at the multiyear-ice station 7 close to the North Pole (Table 3). Station 6 also had the highest megafauna densities along with the MIZ station 4, the lowest densities prevailed at the MIZ station 1 and at the ice-covered stations 8 and 9 (Table 3). There was no statistically significant correlation between estimated taxa richness and megafauna abundance. Individual taxa density with standard deviation can be downloaded from doi.pangaea.de/10.1594/PANGAEA.896638.

**Table 3:** Characteristics of benthic communities and algae aggregations on the seafloor and some environmental and sediment parameters. Total density, total biomass, observed (S) taxa number, Simpson diversity index (1-λ), Shannon diversity index (H'(loge)), Pielou's evenness index (J')) and seafloor algal coverage (%) and algal freshness based on image analysis. Species number in trawl samples based on trawl data. Total number of taxa based on trawl and image data together. Dissolved organic carbon (DOC), total dissolved nitrogen (TDN), chlorophyll a (Chl a) from Rossel et. al. [53]. Sea ice coverage, sea ice thickness and ice age (first (FYI)/multiyear ice (MYI)) from Boetius et al. [71].

Characteristic/Station	1	2	3	4	5	6	7	8	9
Total density (ind. m^-2^) ± SD	1.1 ±0.6	2.9±1.2	3.3 ±1.3	7.8 ±5.3	3.7 ±1.9	4.2 ±1.5	3.7 ±1.2	0.9 ±0.6	1.5 ±0.7
Total biomass (mg ww m^-2^)	240	1020	1640	770	1790	3940	600	210	350
Observed taxa number based on images (S)	26	36	36	35	41	42	19	21	20
Species number based on trawl samples	22	24	17	20	17	24	8	-	-
Taxa number (Total)	38	49	44	46	49	56	23	21	20
Simpson diversity index (1-λ)	0.8	0.8	0.8	0.4	0.7	0.8	0.6	0.7	0.5
Shannon diversity index (H'(loge))	1.8	1.9	2.0	1.0	1.8	1.8	1.2	1.7	1.2
Pielou's evenness index (J')	0.6	0.5	0.5	0.3	0.5	0.5	0.4	0.6	0.4
Seafloor algal coverage (%) ±SD	0	0.03±0.04	1.6±0.4	0.3±0.2	0.5±0.2	0.4±0.6	2.4±0.7	10.3±0.8	0
Algal freshness	absent	mostly fresh	mostly fresh, old white degraded patches present	mostly indistinct old white degraded patches	mostly not old white degraded patches	indistinct very old big white degraded patches	mostly fresh, old white degraded patches present	a lot of small green patches and big brown patches, old white degraded patches present	absent
Dissolved organic carbon (DOC) (μmol/l)	no data	207	317	180	no data	115	147	179	183
Total dissolved nitrogen (TDN) lmol/l	no data	24	31	37	no data	19	24	24	25
Chlorophyll a (Chl a) (μg/CM^3^)	0.07	0.24	0.21	0.22	0.13	0.07	0.08	0.12	0.05
Sea ice coverage (%)	80	80	70	80	60	50	100	100	95
Sea ice thickness (m)	1.0	1.2	0.7	0.7	1.2	0.9	1.2	1.1	1.1
Ice age (first (FYI)/multiyear ice (MYI))	FYI	FYI	FYI	FYI	FYI	FYI/MYI	FYI/MYI	FYI/MYI	FYI/MYI

**Fig 3:**
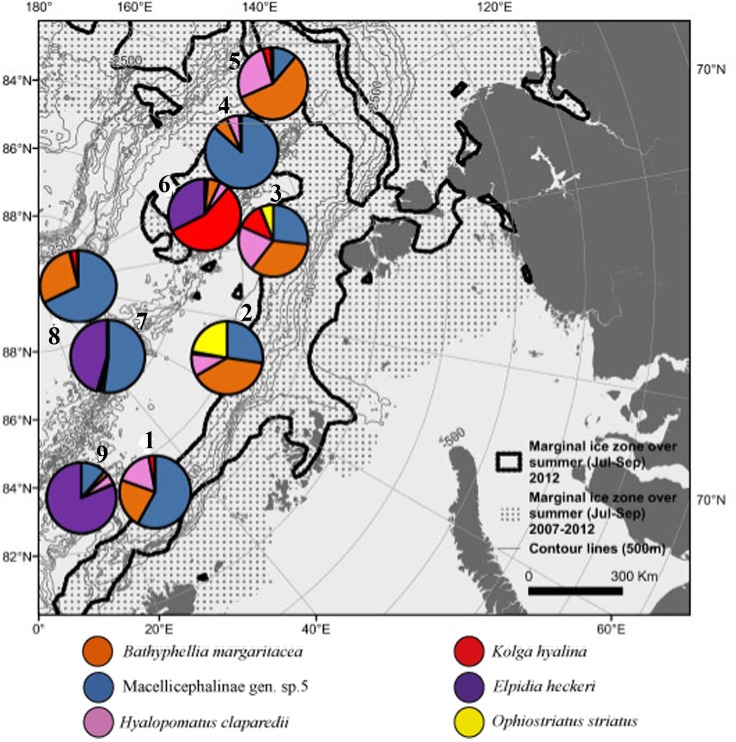
Contribution to abundance (in %) per station of six most abundant taxa.

Images of the most abundant taxa are presented in Fig 2. Percentage of dominance of individual taxa differed between stations (Fig 3, Table 4). Stations 1, 2, 3, 4, 5 and 8 were dominated by the polychaete Macellicephalinae gen. sp. 5 and the actiniarian *Bathyphellia margaritacea*. At stations 7 and 9 the holothurian *Elpidia heckeri* and the polychaete Macellicephalinae gen. sp.5 were dominant. Station 6 was dominated by the holothurians *Kolga hyalina* and *E*. *heckeri*. The highest dominance of a single taxon was at the MIZ station 4 (78%) and at the ice-covered station 9 (71%). Accordingly, evenness was the lowest at station 4 and the highest at the MIZ station 1 and ice-covered station 8. Diversity was the lowest at stations 4 and 9 and the highest at the MIZ station 3 (Table 3).

**Table 4:** Mean density (ind. m^-2^) ± standard deviations and contribution to abundance (in %) of the most abundant taxa at nine stations.

Species	Station								
	1	2	3	4	5	6	7	8	9
Macellicephalinae gen.sp.5	0.5±0.4 42%	0.7±0.6 22%	0.7±0.7 22%	6.1±3.7 78%	0.5±0.7 15%	0.1±0.2 1%	1.7±0.2 47%	0.4±0.4 47%	0.2±0.3 10%
*Bathyphellia margaritacea*	0.2±0.2 16%	1.0±0.56 32%	0.9±0.6 28%	0.6±3.2 7%	1.7±1.0 46%	0.2±0.3 4%	0.0±0.1 0.6%	0.2±0.3 20%	0.0±0.1 2%
*Hyalopomatus claparedii*	0.1±0.2 12%	0.2±0.6 8%	0.6±0.5 17%	0.4±0.4 5%	0.7±0.7 20%	0.2±0.2 3%	0.1±0.2 1%	0.0±0.0 0.3%	0.1±0.2 4%
*Eurycope inermis*	0.1±0.2 9%	0.0±0.0 0.1%	0	0.3±0.6 4%	0.2±0.6 5%	0.8±0.7 18%	0.1±0.2 3%	0.1±0.21 3%	0.1±0.2 3%
*Onisimus leucopis*	0.1±0.2 7%	0.0±0.1 0.6%	0.0±0.1 0.4%	0.0±0.1 0.3%	0.0±0.1 0.5%	0.1±0.1 1%	0.0±0.1 0.7%	0.0±0.1 2%	0.0±0.1 2%
*Kolga hyalina*	0.0±0.1 2%	0	0.3±0.3 10%	0.1±0.2 0.8%	0.1±0.2 3%	1.7±0.8 40%	0.0±0.1 0.6%	0.0±0.1 2%	0.0±0.1 0.3%
*Ascorhynchus abyssi*	0.0±0.1 2%	0.0±0.0 0.2%	0.0±0.1 0.5%	0.2±0.9 2%	0.2±0.3 4%	0.1±0.4 3%	0.0±0.2 0.7%	0.1±0.2 6%	0.0±0.1 1%
*Elpidia heckeri*	0.0±0.0 0.2%	0.0±0.1 0.8%	0.0±0.1 0.3%	0.0±0.1 0.1%	0.0±0.1 0.6%	0.9±0.6 23%	1.5±0.8 41%	0.0±0.0 0.3%	1.1±0.6 71%
*Ophiostriatus striatus*	0	0.5±0.5 18%	0.2±0.3 5%	0	0	0	0	0	0

Dominant taxa are shaded grey.

**Fig 4:**
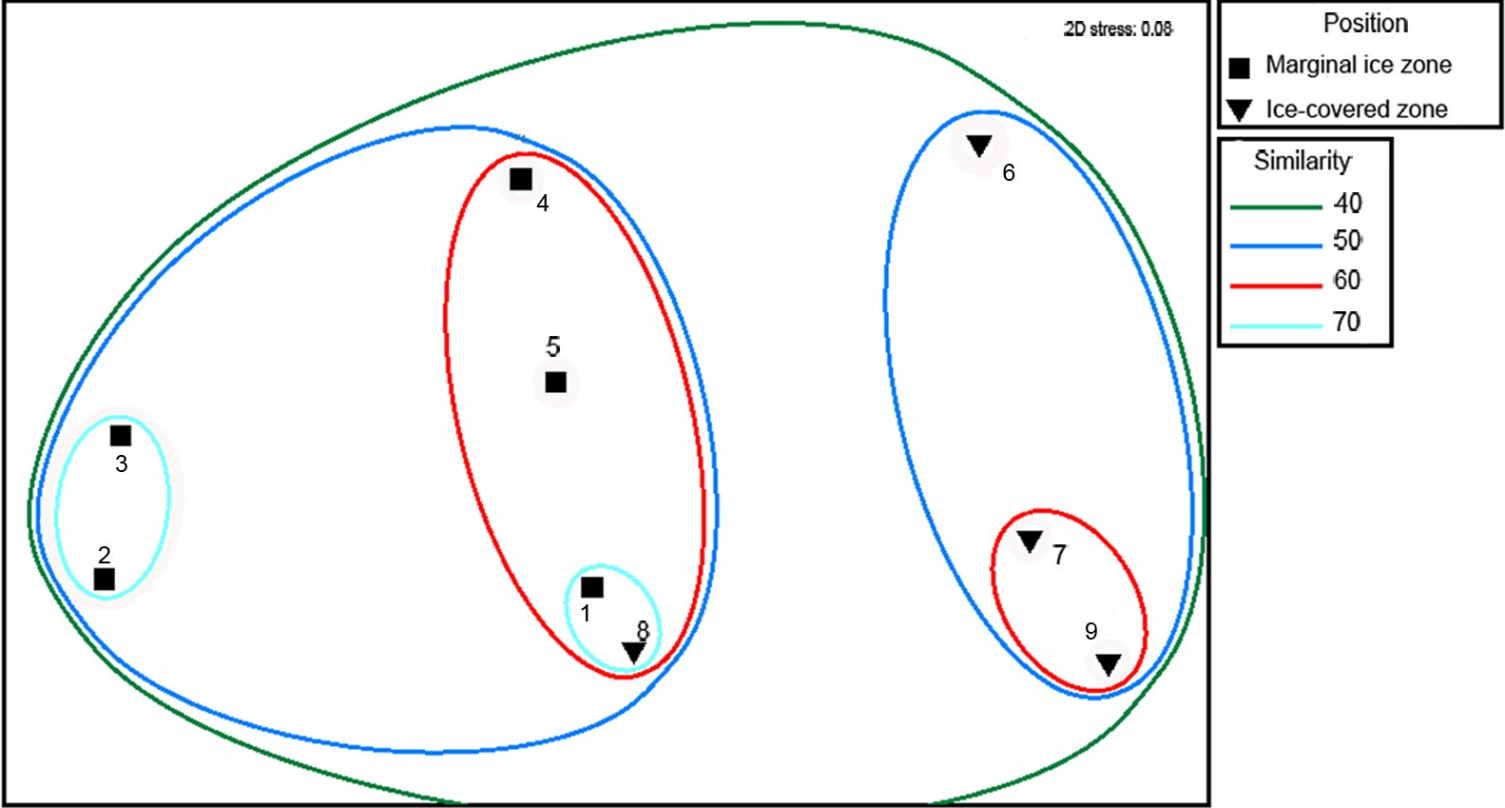
Multidimensional scaling plot for stations based on multivariate Bray-Curtis similarity coefficients for taxa abundance data. Abundance data were standardized to densities and square-root transformed. Similarity (%) is indicated by the coloured lines.

Multi-dimensional scaling of community structure based on taxa abundance data demonstrated considerable dissimilarity between all stations (Bray-Curtis similarity coefficient was around 40%) (Fig 4). Two groups of stations clustered together independent of their origin in the Nansen or Amundsen Basin: three northernmost stations located under mixed first and multi-year ice (stations 6, 7 and 9; group A) and five stations of the marginal ice zone in first year ice (stations 1, 2, 3, 4 and 5; group B). Community structure at station 8 (the northern-most, under multi-year ice) more resembled the composition of MIZ stations. Dissimilarities within group B correlated with the depth: stations 2 and 3 (~3500 m) separated from stations 1, 4, 5 and 8 (>4000 m). Similarity percentages routine (SIMPER) revealed that the average similarity of the group A was based on similar abundance of *Elpidia heckeri*, Macellicephalinae gen. sp. 5 and *Eurycope inermis*. Within the group B the abundance of three species contributed most to similarity: *Bathyphellia margaritacea*, Macellicephalinae gen. sp. 5 and *Hyalopomatus claparedii*. Within the group A station 6 separated from stations 7 and 9. Dissimilarity between these stations was driven mainly by *Kolga hyalina*, *E*. *inermis* and Macellicephalinae gen. sp. 5. Dissimilarity between the two groups within the group B was caused mostly by differences in the abundance of *Ophiostriatus striatus* and Macellicephalinae gen. sp. 5.

#### Biomass distribution

Megabenthos biomass in our study ranged from 0.21 to 3.94 g ww m^-2^. The highest biomass (based on estimations for image data) was at the ice-covered station 6 near Gakkel Ridge. High values were also obtained at the MIZ stations 2, 3 and 5. The lowest biomass was found at the ice-covered station 8 (Table 3).

**Fig 5:**
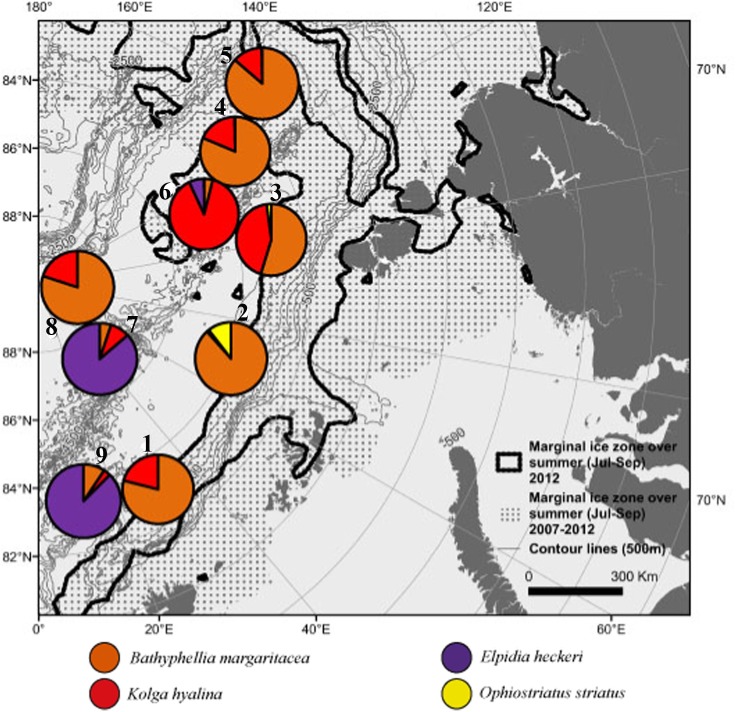
Contribution to biomass (in %) per station of four most abundant taxa.

Taxa dominating the biomass (nine in total) are shown in Fig 5 and Table 5. Three types of megafauna communities were distinguished: dominated by 1) the actiniarian *Bathyphellia margaritacea*, 2) the holothurian *Elpidia heckeri* and 3) the holothurian *Kolga hyalina*. *B*. *margaritacea* dominated at the MIZ stations 1, 2, 3, 4, 5 and at the ice-covered station 8. *K*. *hyalina* was dominant at the ice-covered station 6. *E*. *heckeri* dominated at the ice-covered stations 7 and 9.

**Table 5:** Mean biomass (mg ww m^-2^) and percent of total biomass of the most abundant taxa at nine stations.

Species	Station								
	1	2	3	4	5	6	7	8	9
Macellicephalinae gen. sp.5	7 3%	10 1%	11 1%	90 12%	8 0.4%	1 0%	26 4%	6 3%	2 1%
*Bathyphellia margaritacea*	164 68%	850 83%	842 51%	521 67%	1504 84%	149 4%	21 4%	151 72%	32 9%
*Eurycope inermis*	3 1%	0 0%	0 0%	5 0.6%	6 0.3%	22 1%	4 1%	3 2%	2 0%
*Kolga hyalina*	43 18%	0 0%	659 40%	118 15%	229 13%	3483 88%	44 7%	37 18%	9 3%
*Elpidia heckeri*	1 0%	7 1%	3 0%	2 0.3%	6 0.3%	259 7%	428 71%	1 0%	297 85%
*Ophiostriatus striatus*	0 0%	100 10%	33 2%	0 0%	0 0%	0 0%	0 0%	0 0%	0 0%
Porifera gen. sp.	3 1%	0 0%	57 3%	2 0.3%	4 0.2%	1 0%	0 0%	1 0%	0 0%
*Eurythenes gryllus*	11 5%	12 1%	0 0%	9 1.2%	6 0.3%	7 0%	55 9%	0 0%	0 0%
*Cerianthus* sp.	0 0%	0 0%	11 1%	4 0.5%	11 0.6%	1 0%	8 1%	2 1%	0 0%

Dominant taxa are shaded grey.

#### Algal coverage

**Fig 6:**
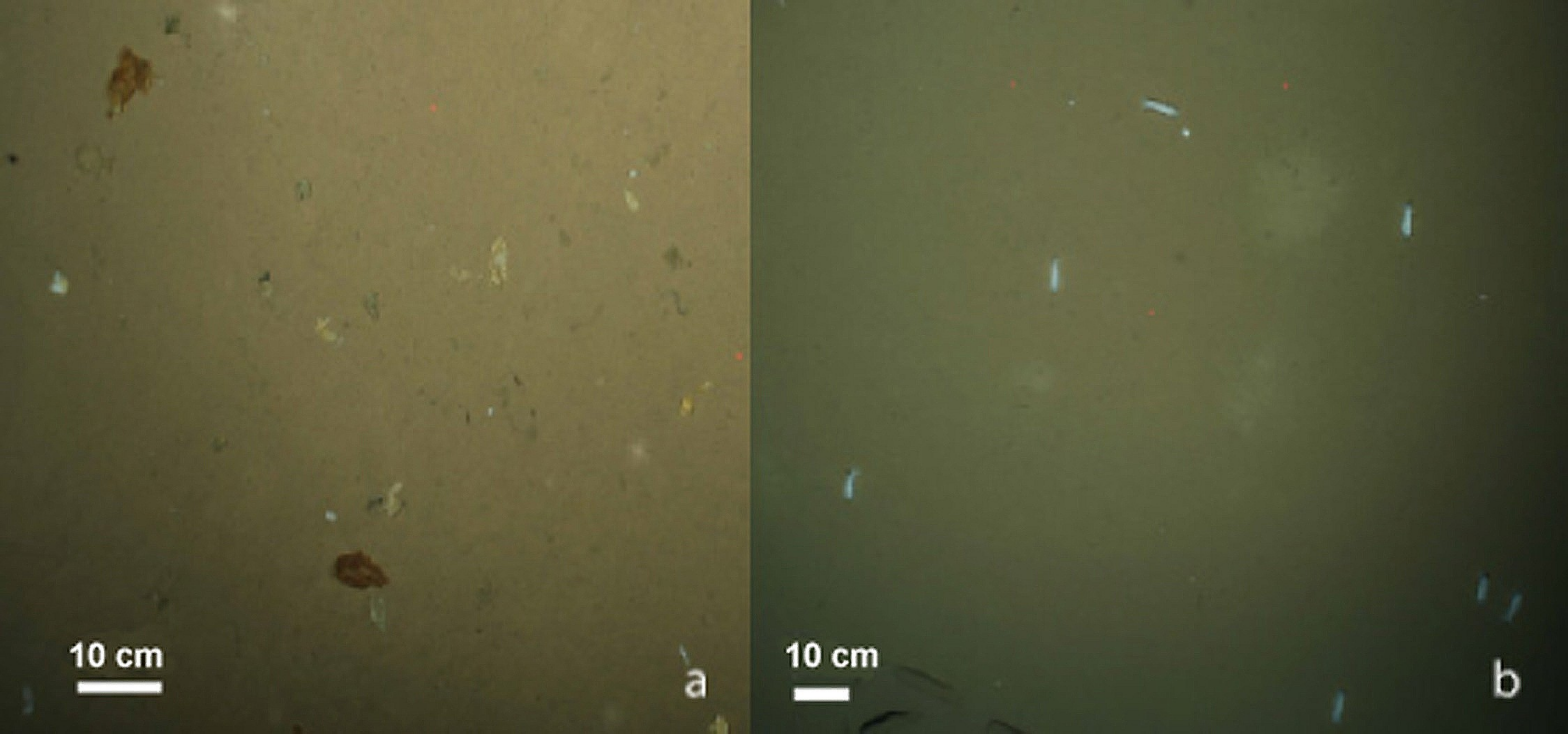
Degree of freshness of ice algae in aggregations at seafloor: (a) greenish-brownish, freshly deposited, (b) whitish-yellowish, mostly degraded diatom falls.

Coverage of seafloor by algal patches varied from almost 0% (stations 1 and 9) to 10±1% (station 8) (Table 3). Patches varied from 5 to 12 cm^2^. At four stations (2,3,7 and 8) the algal falls were mostly fresh (based on visual parameters, Fig 6). At other stations (4,5 and 6) mainly whitish remains of older algal aggregations were common. The oldest remains with no indications of fresh falls were observed at St. 6.

Three benthic species endemic to the Arctic were observed feeding on algae aggregations: the ophiuroid *Ophiostriatus striatus* and the holothurians *Kolga hyalina* and *Elpidia heckeri*. At stations with abundant *K*. *hyalina* (3,4,5 and 6), 10–40% of specimens of this holothurian were associated with algal falls. Maximum values were recorded at stations with mostly fresh (St. 3) or moderately degraded (St. 5) algae. Percent of specimens of another holothurian, *E*. *heckeri*, associated with algae falls was similar: 10–44%, they dominated at St.7 with mostly fresh algae. Percentage of individuals of the ophiuroid *O*. *striatus* associated with algae falls was lower: about 5% at stations 2 and 3.

### Relation between community structure based on images and environmental parameters

**Fig 7:**
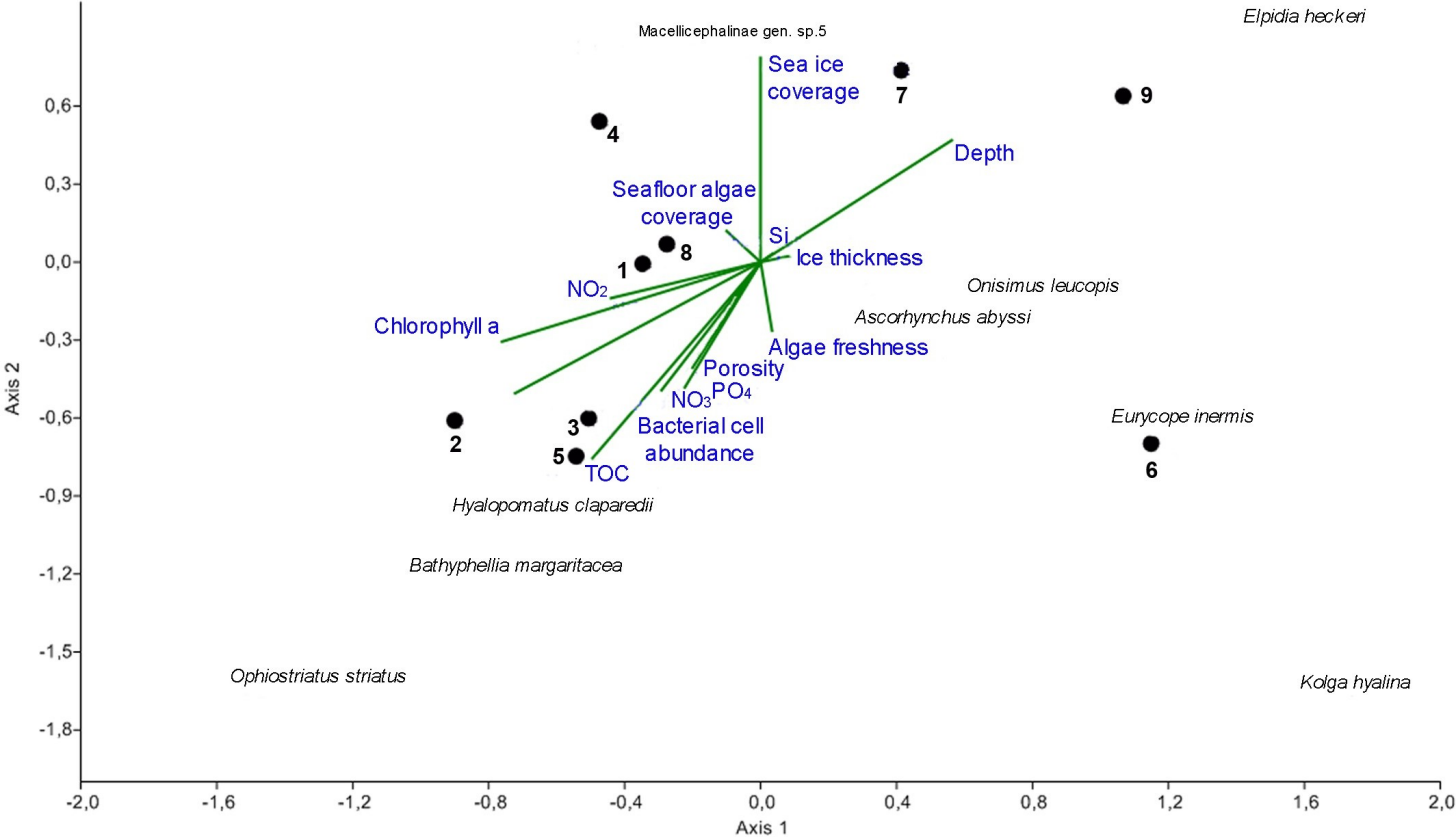
Canonical correspondence analysis (CCA). Explained variance on the first axis—41% and second axis—33%.

Potential individual effects of single environmental factors on megafauna community composition and structure were evaluated. Megafauna communities are known to depend on food supply, here indicated by measured concentrations of chlorophyll a in sediments, as a proxy for the typical background phytodetritus sedimentation, or by the visually detected algal falls as a proxy for the specific 2012 melt-out of ice algae [71]. The latter was assessed based on imagery as algal coverage and categories of algal freshness. Additionally, measured bacterial cell numbers, dissolved organic carbon (DOC) and total organic carbon (TOC) concentrations were included in the analysis, indicative of longer-term variations in food supply (multiyear to decadal time scale). Also sea ice coverage and ice age detected during the cruise, and depth were included in the analysis. Most of Spearman's correlations between environmental parameters and integral community characteristics or individual species density/biomass were not statistically significant. Significant correlations are presented in Table 6. At stations 2, 3, 4, 5 and 6 with the ice coverage <50–80%, the number of taxa was higher than at the northernmost stations (7, 8 and 9) where the ice coverage was 100% (Table 3). This suggests a negative correlation between richness of taxa and the sea ice coverage. Canonical correspondence analysis (Fig 7) revealed a cluster of stations with relatively high densities of the serpulid *Hyalopomatus claparedii* and the actiniarian *Bathyphellia margaritacea* (stations 1,8,2,3 and 5). All of these stations, except 8, were associated with the ice margin and the first-year ice and were characterized by relatively higher concentrations of biogeochemical indicators for food supply. All of these variables showed lower concentrations at stations 6, 7 and 9 located at some distance from the ice edge, and at least partially under thicker multi-year ice and 100% ice cover. They showed a higher densities of the holothurians *Elpidia heckeri* (stations 9 and 7) and *Kolga hyalina* (station 6).

**Table 6:** Spearman's rank correlation between the environmental/sediment parameters and community/species characteristics.

	Number of taxa	*Bathyphellia margaritacea* (density)	*Hyalopomatus claparedii* (density)	*Eurycope inermis* (density)	*Onisimus leucopis* (density)	*Ascorhynchus abyssi* (density)	Total biomass	*Bathyphellia margaritacea* (biomass)
**Sea ice coverage**	-0.94 (0.0006)	p>0.05	p>0.05	p>0.05	p>0.05	p>0.05	-0.73 (0.03)	p>0.05
**TOC**	0.72 (0.03)	0.9 (0.002)	0.73 (0.03)	p>0.05	p>0.05	p>0.05	p>0.05	0.9 (0.002)
**Bacterial cell abundance**	0.74 (0.03)	0.78 (0.01)	0.77 (0.02)	p>0.05	p>0.05	p>0.05	p>0.05	0.78 (0.01)
**Chl a**	p>0.05	0.8 (0.01)	p>0.05	p>0.05	p>0.05	p>0.05	p>0.05	0.82 (0.01)
**Depth**	p>0.05	-0.82 (0.01)	p>0.05	0.78 (0.01)	p>0.05	p>0.05	p>0.05	-0.82 (0.01)
**First/multi year ice**	p>0.05	-0.87 (0.02)	-0.78 (0.03)	p>0.05	p>0.05	p>0.05	p>0.05	-0.87 (0.02)
**DOC**	p>0.05	p>0.05	p>0.05	-0.89 (0.01)	p>0.05	-0.82 (0.02)	p>0.05	p>0.05
**Algal freshness**	p>0.05	p>0.05	p>0.05	p>0.05	p>0.05	0.72 (0.04)	p>0.05	p>0.05
**Percent of seafloor covered by algal**	p>0.05	p>0.05	p>0.05	p>0.05	p>0.05	p>0.05	p>0.05	p>0.05

Correlated pairs with p<0.05 are shaded grey. Chl a–chlorophyll a, DOC—dissolved organic carbon, TOC–total organic carbon. Degree of algal freshness for correlation measurements was evaluated using categories: 0 –absent, 1 –mostly fresh, 2 –mostly old white degraded patches.

However, the effect of the algal coverage as a percent of the total transect area and freshness of algal aggregations was not statistically significantly related to total community structure and taxa abundance/biomass (Table 6). None of the taxa showed a linear correlation with percent of the seafloor covered by algae. In fact, the lowest megafauna density (0.9 ± 0.6) was recorded at St. 8 with the maximum algal coverage (10%), despite the local aggregation of some *Elpidia heckeri* and *Kolga hyalina* feeding on the algal falls.

### Trawl data

In total 2131 specimens of benthic organisms were encountered in the trawl catches from 7 stations.

Fifty one taxa were recorded in trawl samples (32 taxa in the Amundsen Basin and 39 taxa in the Nansen Basin). Nineteen taxa were unique for the Nansen Basin and 12 for the Amundsen Basin. Taxa numbers per trawl varied from 8 (St. 7) to 24 (St. 2). Most abundant in trawl samples were the actiniarian *Bathyphellia margaritacea*, the ophiuroid *Ophiostriatus striatus*, the holothurian *Kolga hyalina*, the polychaete *Anobothrus lauberi* and the bryozoan *Nolella* cf. *dilatata*. The list of identified taxa with abundance and biomass values at each station can be downloaded from doi.pangaea.de/10.1594/PANGAEA.896627 and doi.pangaea.de/10.1594/PANGAEA.896629 correspondingly. Numerous fragments of dead sponges *Caulophacus arcticus* with epifaunal hydrozoans and bryozoans were registered at all stations. Most diverse taxa were polychaetes (9 families), amphipods (7 species, 5 of them of the superfamily Lysianassoidea) and sponges (6 families).

### Differences between trawl and image data

20 taxa were found both in trawl samples and on images (doi.pangaea.de/10.1594/PANGAEA.896618). 31 species was registered only in trawls; 38 only on images.

Overall diversity of Porifera and Hydrozoa in trawl samples was higher in comparison with images. Diversity of isopods in trawl samples was lower (only 1 taxon) compared to images (3 taxa). Also, Macellicephalin polychaetes were diverse and abundant on images, whereas in trawl samples only one species was found.

The most abundant epifaunal species recorded by camera (*Kolga hyalina*, *Elpidia heckeri*, *Bathyphellia margaritacea* and *Ophiostriatus striatus*) were quantitatively underrepresented in trawl catches (first of all *B*. *margaritacea*). This was also the case with mobile organisms (such as amphipods, isopods and swimming polychaetes) caught in very small numbers, potentially due to their ability to escape trawls. At the same time the number of epifaunal hydrozoans, bryozoans and sponges, inhabitants of hard substrata such as stalks of the sponge *Caulophacus*, was underestimated based on images. The infaunal polychaetes caught in trawls were not observed by the OFOS. Altogether, total megafauna density in trawl samples (0.1–0.6 ind m^-2^) was much lower compared to values based on images (0.9–7.8 ind m^-2^).

### Combined trawl and image data, fauna characteristic

At least 89 taxa were recognized in the study area: Seventy one taxon in the Amundsen Basin and 69 in the Nansen Basin. Eighteen taxa were unique for the Nansen Basin and 20 for Amundsen Basin. Total number of taxa per station based on combined image and trawl data varied from 20 (St. 9) to 56 (St. 6). Number of taxa belonging to major taxonomic groups registered on images and trawls is given in Table 2.

Seven generally accepted regions of the ocean were used as a reference in the biogeographic analysis: Arctic Ocean, North Atlantic, South Atlantic, Indian Ocean, North Pacific, South Pacific and Antarctic. Taxa present in all seven regions were classified as cosmopolitan. Endemic species are defined as those that were not reported outside the Arctic Ocean. The Greenland-Scotland Ridge separates the deep Arctic from Atlantic, and shelves of the Chukchi and Bering Seas separate Arctic Basin from the Pacific [92]. Forty two species were identified. Biogeographic analysis revealed that endemicity at the species level was very high: 23 (55%) species are endemic to the Arctic Ocean (S1 Table). Most of the endemic species occur both in Nansen and Amundsen Basins (except of three species known only from the Nansen basin and one species from the Amundsen basin). 24% of species are shared with the deep North Atlantic and 19% are found both in the North Atlantic and North Pacific. Cosmopolitan deep-sea species make up 5%. At the genus level the endemicity was low: 7% among 55 analysed genera are Arctic endemics (S2 Table). About half of the species belong to widespread genera, 48% among them are cosmopolitan and 14% occur in five regions outside the Arctic. The most dominant species (*Bathyphellia margaritacea*, *Kolga hyalina*, *Elpidia heckeri* and *Onisimus leucopis*) in this study were all Arctic endemics.

Our findings expanded the bathymetrical or geographical range of 9 taxa (for details see S3 Table). Maximum depth was extended from bathyal to abyssal for the polychaete *Tubularia regalis*, the bryozoan *Eucratea loricate* and the asteroid *Tylaster wyllei*. Known depth was also increased for the polychaete *Hyalopomatus claparedi* and the decapod *Bythocaris curvirostris*. Cnidarian *Bouillonia* sp., Echiurida fam. indet. and Enteropneusta fam. indet. were for the first time recorded in the Central Arctic. The cnidarian *Oceanactis bursifera* was found in our study the first time since the description of this species in 2000.

## Discussion

The present study aimed at providing quantitative data on the megafauna distribution and community structure in the ice-covered deep Nansen and Amundsen Basins of the Central Arctic. These basins show substantial change in sea ice cover owing to warming, yet little is known how these changes are reflected in phytodetritus export and responses by the deep-sea benthos.

We were able to identify in this area 89 benthic taxa in total, based on images and trawl samples, and quantified their distribution to test the effect of ice cover and other spatial differences. Overall the examined fauna includes mainly the Arctic endemics or the Arctic-North Atlantic species belonging to genera widely distributed in the World Ocean. These results agree with trends shown by Mironov et al. [56].

### Sea ice, food supply and other factors structuring megafauna communities in the Eurasian basins

Stations investigated in the present study showed a substantial variation in the megafauna community structure, with a significant clustering of stations in relation to their proximity to the ice margin, which in 2012 (the year of study) had shifted northwards due to an unprecedented sea-ice minimum. Total biomass was negatively correlated with sea ice coverage and distance from the ice edge. In earlier studies of pan-Arctic deep seas it was shown that the density and biomass of mega-, macro- and meiofauna increased in the marginal ice zone [14, 15, 55, 93, 94].

This substantial effect of ice-cover on community composition appears to be mostly related to variations in food supply. Several biogeochemical variables corresponding to the input of phytodetrital material on the seafloor demonstrate higher fluxes closer to the MIZ than at the northernmost stations [71, 53]. Because of low bioturbation rate and relatively low degradation rate in polar waters, it is assumed that variables such as chlorophyll a pigments, total and dissolved organic carbon and nutrient concentrations reflect differences in food supply over the time scale of years to decades [47, 53].

Relationship between the composition and structure of megafauna communities and the observed biogeochemical indicators of food supply was not linear. A tendency of increasing abundance with increasing food supply was shown for the polychaete *Hyalopomatus claparedii* and the actiniarian *Bathyphellia margaritacea*. On the other hand, the abundance of holothurians *Elpidia heckeri* and *Kolga hyalina* declined with increasing food supply. Notably the dominant species differ in their trophic modes: actiniarians and polychaetes are suspension feeders, whereas holothurians are deposit feeders. Significance of correlations increased when the biomass values were used instead of abundance.

Depth also had an effect on biomass in our study. Though stations in our study were located in the abyssal zone (from 3400 m to 4400 m) we observed a biomass decrease with increasing depth fitting the globally predicted trend [95, 96]. Megabenthos biomass in the Nansen and Amundsen Basins in our study was close to estimates of the macrobenthos biomass in the deep-sea Arctic [45, 46] and close to predicted calculated values for the Arctic fauna at depths 3500–4000 m [96].

The present study also revealed a negative correlation between the number of taxa and the sea ice coverage, the latter increasing towards the North Pole. Earlier Wƚodarska-Kowalczuk et al. [55] demonstrated that the species richness of the deep-sea Arctic fauna in general declines towards the North Pole. Our work indicates an important effect of ice-cover and food supply on diversity of megafauna communities.

### Comparison with megafauna distribution in other Arctic regions

Relatively high proportions of suspension feeding anthozoans at HAUSGARTEN [14], in Canada [40], Nansen and Amundsen Basins (present study) at depths 3000–4300 m is a characteristic pan-Arctic feature. In other regions such suspension feeders are common at mid-slope depths [97].

In the present study unidentified swimming macellicephalin polychaetes were relatively abundant at depths 3570–4380 m. Dominance of unidentified swimming polychaetes was previously reported from the Canada Basin based on ROV observations (2800 m) [39].

Holothurians often dominate megafauna in the deep-sea [98], as also shown in our study. At the HAUSGARTEN observatory in the Fram Strait, *Elpidia heckeri* dominated the abundance at depths 5333–5404 m and *Kolga hyalina* at 2609–2629 m [14]. The first depth is somewhat deeper and the second shallower compared to our results. Uneven distribution of holothurians at the abyssal seafloor, similar to our results, was demonstrated for the Canada Basin [39, 40].

The ophiuroid *Ophiostriatus striatus* was also numerous but not the dominant species at two shallower stations in the Nansen Basin. Ophiuroids often dominate the Arctic shelf epifauna reaching peak densities of several hundred ind. m^-2^ [72, 5, 8].

### Relationship between the ice algae aggregations on the seafloor and megafauna

Extensive aggregates of the sea-ice diatom *Melosira arctica* on the seafloor in the Arctic abyss were observed for the first time on the ARK-XXVII/3 expedition in 2012 [71]. This observation combined with the sediment trap data, benthic respiration rates and oxygen profiles in the sediment led to the hypothesis that extensive deposition of sea-ice algae to the deep seafloor was a consequence of rapid ice-melt in 2012, a recent phenomenon for the deep-sea Arctic. Image analysis and investigation of the gut content of selected species showed that only a few large mobile megafauna species, such as the ophiuroid *Ophiostriatus striatus* and the holothurians *Kolga hyalina* and *Elpidia heckeri*, accumulated on ice-algae patches for feeding [71].

In the present study we quantified specific responses and associations with ice-algal falls. As *Kolga hyalina* and *Elpidia heckeri* dominated the megafauna in the same year when substantial algal falls occurred, we suggest that these species may be indicative of melt events and their abundance may increase significantly with recurring strong melt events, as observed recently during Arctic summer. Studies in different world ocean regions (North Atlantic and North Pacific, Antarctica and California coast) have demonstrated that abyssal megafauna, mainly holothurians, is able to react rapidly to substantial changes in food supply [99, 100, 101, 102, 103, 104, 105]. However, we still found only weak evidence for a direct relationship between the megafauna abundance and biomass and the density of algal aggregations at the seafloor or the state of the algae (fresh vs. degraded). This supports the hypothesis that such algal food falls in the deep Arctic Basins are a recent phenomenon [71], and that it may take time for the fauna to adapt and to exploit such food falls.

In our study the elpidiid *Kolga hyalina* occurred in the highest density at St. 6 with only old degraded algal deposits, indicating that the algal flux in that area took place before June [71]. High density of sea cucumbers at this station might indicate that algal falls may have occurred the year before and potentially provided energy for a higher population density. *K*. *hyalina* is benthopelagic species, able to swim in the near-bottom water layer [40, 99, 100, present OFOS observations]. This feature may help species of *Kolga* to respond rapidly to seasonal accumulations of organic matter at the seafloor. To fully understand the role of the ice-algae supply to the abyssal seafloor in the Arctic and the effect of this phenomenon on the abyssal benthic life, further studies are required.

### Abyssal megafauna communities and possible impacts of climate change

Models predict continuing rapid warming of climate in the Northern Hemisphere causing the reduction of the sea-ice extent and thickness, including ice-free summers in the Arctic Ocean [59, 106]. Different scenarios of increasing or decreasing of photosynthesis based primary production have been discussed, as both sunlight and nutrient supply limit Arctic productivity and export flux [64, 65, 66, 67, 68]. Our study has revealed the relationship between the ice-cover, food supply and structure of deep-sea megafauna communities.

Variations in the megabenthos community structure between stations in our study may reflect short and long-term variations in phytodetritus flux to the seafloor. This flux is likely to depend on distance from the sea-ice margin and ice thickness. Hence, it can be expected that further warming and sea ice retreat will affect carbon flux to the abyssal and thereby the biodiversity and distribution of Arctic fauna. Unfortunately, no quantitative baseline data are available for the Central Arctic megabenthos before 2000. Assessment of epibenthic megafauna of the HAUSGARTEN area from 2000 to 2012 showed significant changes in relative abundances of megafauna species that were related to variations in food supply with time, apparently linked to dynamics in sea ice cover and hydrography [15, 20].

Present study emphasizes the need for accumulating quantitative data on seasonal and annual variations of the Central Arctic megabenthos, to detect ecosystem changes in the deep-sea. According to our comparison of image-based and trawl-based data, it seems more useful for long-term monitoring to use imaging along standard transects: image data allow for a more correct assessment of monitored species density. Present work contributes to baseline data for the Nansen and Amundsen Basins, and to the first assessment of deep-sea benthos responses to sea-ice algae food falls.

## Conclusion

Our study on the composition and structure of megabenthos communities in different areas of the Eastern Central Arctic Basin combining quantitative photographic surveys with trawl sampling provides quantitative information of the dominant megafauna of the ice-covered basins and key factors structuring the distribution of abyssal megafauna in the Central Arctic. Three types of megafauna communities were distinguished: dominated by 1) the actiniarian *Bathyphellia margaritacea*, 2) the holothurian *Elpidia heckeri* and 3) the holothurian *Kolga hyalina*. Variations in megafaunal abundance were first of all related to the proximity to the marginal ice zone. Stations closer to the ice margin under first-year ice were characterized by relatively high densities and biomasses of *B*. *margaritacea* and relatively high food supply to the seafloor indicated by several biogeochemical variables. Stations located closer to the North Pole under the multi-year ice showed relatively low food supply, but relatively high densities and biomasses of holothurians *E*. *heckeri* and *K*. *hyalina* feeding on fresh algal falls of the colonial sea-ice diatom *Melosira arctica*. In case extensive algal food falls to the seafloor become regular as a result of increasingly frequent sea-ice melt events, the abundance of mobile deposit-feeding megafauna, such as elpidiid holothurians and ophiuroids, in the abyssal Central Arctic may rise significantly. Our data provide a baseline for long-term monitoring of the seafloor of the changing deep-sea Arctic Ocean.

## Supporting information

S1 Table Characteristics of biogeographic distribution of species founded in the OFOS photographic survey and collected by Agassiz trawl during POLARSTERN cruise PS80 (ARK-XXVII/3, IceArc) to the Central Arctic Ocean in August and September 2012 (DOI) (Supplementary, PDF). (PDF)

S2 Table Characteristics of biogeographic distribution of genus founded in the OFOS photographic survey and collected by Agassiz trawl during POLARSTERN cruise PS80 (ARK-XXVII/3, IceArc) to the Central Arctic Ocean in August and September 2012 (DOI) (Supplementary, PDF). (PDF)

S3 Table New taxonomic findings and depth extension for megafauna founded in the OFOS photographic survey and collected by Agassiz trawl during POLARSTERN cruise PS80 (ARK-XXVII/3, IceArc) to the Central Arctic Ocean in August and September 2012. Sample methods, location, depth and previously known distribution and depth range are shown (DOI) (Supplementary, PDF).
(PDF)
