# Supplementary material for: Deep-sea megabenthos communities of the Eurasian Central Arctic are influenced by ice-cover and sea-ice algal falls
Source: PLoS One. 2019 Jul 16;14(7):e0211009. doi: 10.1371/journal.pone.0211009 (PMC6634375; doi:10.1371/journal.pone.0211009)
Supplement: S1 Table — (PDF) [file pone.0211009.s001.pdf]

S5 Table. Characteristics of biogeographic distribution of species founded in the OFOS photographic survey and collected by Agassiz trawl during POLARSTERN cruise PS80 (ARK-XXVII/3, IceArc) to the Central Arctic Ocean in August and September 2012.

1 - Central Arctic, depths >500 m;

2 - deep Norwegian and Greenland Seas, depths >500 m;

3 - Arctic seas and also Greenland Sea, depth <500 m;

4 - Shelf depths (0-500 m) of Eastern and Western Atlantic; in the East Atlantic from Finistere on the South to Nordkapp on the North, in the West Atlantic from Cape Hatteras to Newfoundland (from Golikov et al., 1990, modified [1]);

5 - Shelf depths (0-500 m) of East and West Pacific from 38ºN on the south to Bering Strait (from Golikov et al., 1990 [1]);

6- North Atlantic ocean, depths >500m;

7- North Pacific ocean, depths >500m;

8- Atlantic ocean except regions 4 and 6;

9- Pacific ocean except regions 5 and 7;

10 - Indian Ocean;

11 - Antarctic, to south of 60ºS.

Species/Region	1	2	3	4	5	6	7	8	9	10	11	Source
Caulophacus arcticus	+	+				+						[2]
Thenea abyssorum	+	+										[3]
Geodia phlegraei	+	+	+	+		+						[4]
Craniella cranium	+		+	+				+				[5]
Bathyphellia margaritacea	+	+										[6]
Oceanactis bursifera	+											[7]

Symplectoscyphus tricuspidatus	+	+	+	+	+			+	+			[8]
Tubularia regalis	+		+	+				+		+		[9]
Sertularia tenera	+		+	+	+							[9]
Hyalopomatus claparedii	+	+	+									[10]
Potamethus malmgreni	+	+										[11]
Bathypolaria carinata	+											[12, 13]
Chauvinelia arctica	+	+										[14]
Aglaophamus malmgreni	+	+	+	+	+							[12, 13]
Ophelina opisthobranchiata	+	+										[15]
Anobothrus laubieri	+	+	+									[16]
Siboglinum ekmani	+	+		+		+						[17]
Katadesmia kolthoffi	+	+										[18]
Yoldiella annenkovae	+			+								[19]
Yoldiella frami	+	+										[19]
Cuspidaria centobi	+	+										[18]
Mohnia danielsseni	+	+										[20]
Turrisipho lachesis	+	+	+			+						[20]
Cirroteuthis muelleri	+	+	+		+	+		+				[21, 22]
Eurycope inermis	+	+	+	+								[23]
Eurythenes gryllus	+	+				+	+	+	+	+	+	[24]
Onisimus leucopis	+	+										[25]
Halirages qvadridentatus	+	+	+			+						[25]
Tryphosella abyssalis	+	+	+									[25]
Centromedon typhlops	+	+										[25]
Leucothoe uschakovi	+	+										[25]
Bythocaris curvirostris	+	+										[26]

Bythocaris payeri	+	+	+	+								[26]
Hymenodora glacialis	+	+				+	+	+				[27]
Ascorhynchus abyssi	+	+				+						[28]
Pseudosagitta maxima	+	+	+	+	+	+	+	+	+	+	+	[29]
Eucratea loricata	+	+	+	+	+			+				[30, 31]
Tylaster willei	+	+										[32]
Elpidia heckeri	+	+										[33]
Kolga hyalina	+	+										[33]
Ophiostriatus striatus	+	+										[34]
Bathycrinus carpenterii	+	+										[35]

References

1. Golikov AN, Dolgolenko MA, Maximovich NV, Scarlato OA. Theoretical approaches to marine biogeography. Mar Ecol Prog Ser 1990; 63: 289-301.
2. Tabachnik KR. Deep-sea fauna of European seas: An annotated species check-list of benthic invertebrates living deeper than 2000 m in the seas bordering Europe. Porifera. Invertebrate Zool. 2014; 11 (1): 231–239.
3. Barthel D, Tendal OS. The sponge association of the abyssal Norwegian Greenland Sea: Species composition, substrate relationships and distribution. Sarsia [Internet]. 1993; 78: 83–96.
4. GBIF.org (29th December 2018) GBIF Occurrence Download https://doi.org/10.15468/dl.iyobdu
5. Dinn C, Leys SP. Field Guide to Sponges of the Eastern Canadian Arctic. A field and laboratory identification guide. Ver. 1.0. 2018. Edmonton, Canada.
6. Sanamyan NP, Cherniaev ES, Sanamyan KE. *Bathyphellia margaritacea* (Cnidaria: Actiniaria): the most northern species of the world. Polar Biol [Internet]. 2009; 32: 1245–1250. Available from: https://doi.org/10.1007/s00300-009-0685-3

7. Riemann-Zürneck K. *Oractis bursifera* sp. nov., an Arctic deep-sea anemone with peculiar invaginations of its oral disc (Cnidaria: Actiniaria). Polar Biol [Internet]. 2000; 23: 604–608. Available from: https://doi.org/10.1007/s003000000127
8. GBIF.org (29th December 2018) GBIF Occurrence Download https://doi.org/10.15468/dl.qn24zh
9. Stepanjants SD. Hydrozoa of the Eurasian Arctic Seas. In: Herman Y., editor. The Arctic Seas. 1989. Springer, Boston, MA
10. Kupriyanova EK, Jirkov IA. Serpulidae (Annelida, Polychaeta) of the Arctic Ocean. Sarsia [Internet]. 1997; 82: 203–236. Available from: https://doi.org/10.1080/00364827.1997.10413651
11. Jirkov IA, Leontovich MK. Biogeography of Polychaeta of the Eurasian North Polar Basin. Invertebrate Zool. 2012; 9 (1): 41-51.
12. Jirkov IA. Polychaeta of the Arctic Ocean. Moscow: Yanus-K Press. 632 p., 2001
13. Budaeva NE, Jirkov IA, Savilova TA, Paterson GLJ. Deep-sea fauna of European seas: An annotated species check-list of benthic invertebrates living deeper than 2000 m in the seas bordering Europe. Polychaeta. Invertebrate Zool. 2014; 11 (1): 217–230
14. Salazar-Vallejo SI, Gillet P, Carrera-Parra LF. Revision of *Chauvinelia*, redescriptions of *Flabelliseta incrusta*, and *Helmetophorus rankini*, and their recognition as acrocirrids (Polychaeta: Acrocirridae). J Mar Biol Assoc U.K. 2007; 87: 465-477 doi:10.1017/S0025315407054501
15. Kongsrud JA, Bakken T, Oug E. Deep-water species of the genus *Ophelina* (Annelida, Opheliidae) in the Nordic Seas, with the description of Ophelina brattegardi sp. nov. Ital J Zool [Internet]. 2011; 78: 95–111. Available from: https://doi.org/10.1080/11250003.2011.606658
16. Jirkov IA. Revision of Ampharetidae (Polychaeta) with modified thoracic notopodia. Invertebrate Zool. 2009; 5 (2): 111–132.
17. Webb M. A redescription of *Siboglinum ekmani* jÄgersten (pogonophora). Sarsia [Internet]. 1964; 15: 37–47. Available from: https://doi.org/10.1080/00364827.1964.10409527
18. Janssen R, Krylova EM. Deep-sea fauna of European seas: An annotated species check-list of benthic invertebrates living deeper than 2000 m in the seas bordering Europe. Bivalvia. Invertebrate Zool. 2014; 11 (1): 43–82.
19. Warén A. Taxonomic comments on some protobranch bivalves from the northeastern Atlantic. Sarsia [Internet]. 1989; 74: 223–259. Available from: https://doi.org/10.1080/00364827.1989.10413432
20. Golikov AN. Shell-bearing gastropods of the Arctic. M., Colus, 108 p., 1994.

21. Collins MA. Cirrate octopods from Greenland and Iceland waters. J Mar Biol Assoc U.K. [Internet]. 2002; 82: 1035–1036. Available from: https://www.cambridge.org/core/article/cirrate-octopods-from-greenland-and-iceland-waters/CA3C5F9F5F066D755F406D9574651EA3
22. Nesis KN. West-Arctic and East-Arctic distributional ranges of cephalopods. Sarsia [Internet]. 2001; 86: 1–11. Available from: https://doi.org/10.1080/00364827.2001.10420456
23. Wilson G. Systematics of a species complex in the deep-sea genus *Eurycope*, with a revision of six previously described species (Crustacea, Isopoda, Eurycopidae). Bulletin of the Scripps Insitution of the Oceanography of the University of California La Jolla, California. Cox CS, Vincent ES, Fleminger A, Rosenblatt RH, editors. Uneversity of California Press, Ltd, London, England. 1983.
24. Stoddart HE, Lowry JK. The deep-sea lysianassoid genus *Eurythenes* (Crustacea, Amphipoda, Eurytheneidae n. fam.). Zoosystema. 2004; 26 (3): 425-468.
25. Gurjanova EF. Bokoplavy Morej SSSR i Sopredel'nykh Vod (Amphipoda-Gammaridea) [Amphipods of the Seas of the USSR and Adjacent Waters (Amphipoda, Gammaridea)]. In: Opredeliteli po Faune SSSR [Identification Fauna USSR]. 1951. p. 1031.
26. Sokolov V. Deep-sea shrimps of the genus *Bythocaris* GO Sars in the collections of Russian museums, with the description of a new species (Crustacea: Decapoda: Hippolytidae). ZOOLOGISCHE MEDEDEELINGEN. 2000; 403-468.
27. Havens AD, Rork WL. *Hymenodora glacialis* (Decapoda: Natantia) from the Arctic Basin. Bull South Calif Acad Sci. 1969; 68: 19–29.
28. Turpaeva EP, Raiskiy AK. Deep-sea fauna of European seas: An annotated species check-list of benthic invertebrates living deeper than 2000 m in the seas bordering Europe. Pycnogonida. Invertebrate Zool. 2014; 11 (1): 240–247.
29. GBIF.org (02 January 2019) GBIF Occurrence Download https://doi.org/10.15468/dl.mosumn
30. Denisenko NV. Deep-sea fauna of European seas: An annotated species check-list of benthic invertebrates living deeper than 2000 m in the seas bordering Europe. Bryozoa. Invertebrate Zool. 2014; 11 (1): 89–98
31. GBIF.org (02 January 2019) GBIF Occurrence Download https://doi.org/10.15468/dl.yfvhqz
32. Dilman AB. Deep-sea fauna of European seas: An annotated species check-list of benthic invertebrates living deeper than 2000 m in the seas bordering Europe. Asteroidea. Invertebrate Zool. 2014; 11 (1): 25–4.

33. Rogacheva A. Revision of the Arctic group of species of the family Elpidiidae (Elasipodida, Holothuroidea). Mar Biol Res. 2007;3: 367–396.
34. Smirnov IS, Piepenburg D, Ahearn C, Juterzenka KV. Deep-sea fauna of European seas: An annotated species check-list of benthic invertebrates living deeper than 2000 m in the seas bordering Europe. Ophiuroidea. Invertebrate Zool. 2014; 11 (1): 192–200.
35. Mironov AN, Améziane N, Eléaume MP. Deep-sea fauna of European seas: An annotated species check-list of benthic invertebrates living deeper than 2000 m in the seas bordering Europe. Crinoidea. Invertebrate Zool. 2014; 11 (1): 112–119.
