# Supplementary material for: Deep-sea megabenthos communities of the Eurasian Central Arctic are influenced by ice-cover and sea-ice algal falls
Source: PLoS One. 2019 Jul 16;14(7):e0211009. doi: 10.1371/journal.pone.0211009 (PMC6634375; doi:10.1371/journal.pone.0211009)
Supplement: S2 Table — (PDF) [file pone.0211009.s002.pdf]

S6 Table. **Characteristics of biogeographic distribution of genus founded in the OFOS photographic survey and collected by Agassiz trawl during POLARSTERN cruise PS80 (ARK-XXVII/3, IceArc) to the Central Arctic Ocean in August and September 2012.**

**Genus/Region**	**North Atlantic**	**South Atlantic**	**North Pacific**	**South Pacific**	**Indian Ocean**	**Antarctica**
Caulophacus						
Thenea	+	+	+	+	+	
Sycon						
Geodia	+	+	+	+	+	
Craniella						
Asbestopluma						
Bathyphellia			+			+
Oceanactis			+	+		
Cerianthus	+	+	+	+	+	
Keratoisis						
Garveia						
Abietinaria	+		+		+	
Symplectoscyphus						
Tubularia	+	+	+	+		+
Bouillonia		+				+
Eudendrium						
Sertularia						
Bathykorus						
Hyalopomatus	+		+	+		+
Potamethus	+	+	+			+
Bathypolaria						
Chauvinelia	+					
Aglaophamus						

Abyssoninoe	+	+		+	+	+
Ophelina						
Anobothrus						
Terebellides						
Siboglinum	+	+	+			
Echiurus						
Dinonemertes			+			
Uniporus	+					
Katadesmia						
Yoldiella						
Cuspidaria						
Mohnia	+	+	+			
Turrisipho	+					
Cirroteuthis	+	+	+	+		
Eurycope						
Eurythenes						
Onisimus						
Halirages						
Halice	+	+	+	+		+
Tryphosella						
Centromedon	+	+	+			
Hippomedon						
Leucothoe						
Bythocaris	+			+		
Hymenodora						
Eucratea	+		+			
Nolella	+	+	+	+	+	

Elpidia						
Kolga						
Ophiostriatus	+	+	+		+	+
Bathycrinus						
Tylaster						
