# Supplementary material for: Deep-sea megabenthos communities of the Eurasian Central Arctic are influenced by ice-cover and sea-ice algal falls
Source: PLoS One. 2019 Jul 16;14(7):e0211009. doi: 10.1371/journal.pone.0211009 (PMC6634375; doi:10.1371/journal.pone.0211009)
Supplement: S3 Table — Sample methods, location, depth and previously known distribution and depth range are shown (DOI) (Supplementary, PDF). (PDF) [file pone.0211009.s003.pdf]

S7 Table. New taxonomic findings and depth extension for megafauna founded in the OFOS photographic survey and collected by Agassiz trawl during POLARSTERN cruise PS80 (ARK-XXVII/3, IceArc) to the Central Arctic Ocean in August and September 2012. Sample methods, location, depth and previously known distribution and depth range are shown.

Taxon	Findings during ARK XVII-3				Previously known distribution	Previously known depth range (m)
	OFOS		Trawl			
	Location (station #)	Depth (m)	Location (station #)	Depth (m)		
Oceanactis bursifera (Riemann-Zurneck, 2000) Cnidaria, fam. Oractinidae	Nansen Basin (st. 1,2)	3468-3575	-	-	Single record in the Central Arctic (Amundsen Basin, Laptev Sea) [1, 2]	3012-3028
Tubularia regalis (Boeck, 1860) Cnidaria, fam. Tubulariidae	Amundsen Basin (st.5)	4032-4041	-	-	Deep-sea Greenland and Norwegian Basins [3];<br>Arctic shelf (Oslo fjord – [4];<br>Central Arctic [5]	50-2340
Bouillonia sp. Cnidaria, fam. Tubulariidae	-	-	Nansen Basin (st.3)	3575	Laptev Sea (*Bouillonia* another species) [6];<br>Slope off Svalbard [6];<br>South Atlantic [6];<br>Antarctic [6]	
Hyalopomatus claparedi (Marenzeller, 1878) Annelida, fam. Serpulidae	Nansen Basin (st. 1,2,3,9)	3468-4066	Nansen Basin (st. 1,2,3)	3471-4013	Deep-sea Greenland and Norwegian Basins (Greenland slope, [7];<br>Arctic shelf (Greenland slope, [7];<br>Central Arctic [7]	212-3622
	Amundsen Basin (st.4,5,6,7,8)	4033-4383	Amundsen Basin (st.4,5,6)	4037-4354		

Echiurida fam indet.<br>Annelida	-	-	Amundsen Basin (st.4,6)	4157-4354	Only *Hamingia arctica* is known from Fram Strait [8]	2500
Bythocaris curvirostris<br>(Kobjakova, 1957)<br>Arthropoda, fam. Bythocarididae	Nansen Basin (st. 2,3)	3468-3575	Nansen Basin (st.2)	3471	deep-sea Greenland and Norwegian Basins [9];<br>Central Arctiic [10]	2730-3440
			Amundsen Basin (st. 5)	4039		
Eucratea loricata<br>(Linnaeus, 1758)<br>Bryozoa, fam. Eucrateidae	Nansen Basin (st. 2)	3468	Amundsen Basin (st. 4)	4159	Deep-sea Greenland and Norwegian Basins [11];<br>Arctic Shelf [11];<br>Central Arctic (Laptev Sea slope, [5];<br>North Atlantic [11];<br>Atlantic boreal zone [11];<br>Pacific boreal zone [11]	3-2340
	Amundsen Basin (st.5,6)	4032-4354				
Tylaster wyllei<br>(Danielssen & Koren, 1881)<br>Echinodermata, fam. Poraniidae	-	-	Nansen Basin (st. 3)	3575	Deep-sea Greenland and Norwegian Basins [12];<br>Arctic slope [12];<br>Arctic shelf (St. Anna Trough, [13])	79-2920
Enteropneusta fam indet.<br>Hemichordata	Amundsen Basin (st.4,5,6)	4032-4354	-	-	Fram Strait (different family) [14]	2363-2515

References

1. Riemann-Zürneck K. *Oractis bursifera* sp. nov., an Arctic deep-sea anemone with peculiar invaginations of its oral disc (Cnidaria: Actiniaria). Polar Biol. 2000; 23: 604-608.

2. Sanamyan NP. New Record of *Oceanactis diomedeae* (Cnidaria: Actiniaria: Oractiidae) and Systematic Position of the Genera *Oceanactis* and *Oractis*. Species Divers. 2003; 8: 93-101.

3. Schuchert P. The European athecate hydroids and their medusae (Hydrozoa, Cnidaria): Capitata part 2. Revue suisse de Zoologie. 2010; 117 (3): 337-555.

4. Christiansen BO. The hydroid fauna of the Oslo Fjord in Norway. Norwegian Journal of Zoology. 1972; 20: 279-310.

5. Sirenko B, Denisenko S, Deubel H, Rachor E. Deep water communities of the Laptev Sea and adjacent parts of the Arctic Ocean. Fauna and the ecosystems of the Laptev Sea and adjacent deep waters of the Arctic Ocean. Explorations of the fauna of sea. St. Petersburg: Zoological Institute of Russian Academy of Sciences. 2004; 54(62): 28-73.

6. Svoboda A, Stepanjants SD, Ljubenkov J. The genus *Bouillonia* (Cnidaria: Hydrozoa: Anthoathecata). Three species from the northern and southern hemispheres with a discussion of bipolar distribution of this genus. Zool Med Leiden. 2006; 80-4 (14): 185-206.
7. Kupriyanova EK, Zhirkov IA. Serpulidae (Annelida, Polychaeta) of the Arctic Ocean. Sarsia. 1997; 82: 203-236.
8. Meyer KS, Bergmann M, Soltwedel T. Interannual variation in the epibenthic megafauna at the shallowest station of the HAUSGARTEN observatory (79_ N, 6_ E). Biogeosciences. 2013; 10: 3479-3492.

9. Sokolov V. Deep-sea shrimps of the genus *Bythocaris* GO Sars in the collections of Russian museums, with the description of a new species (Crustacea: Decapoda: Hippolytidae). Zool Meded. 2000; 74: 403-468.

10. Vassilenko SV, Petryashov VV, editors. Illustrated keys to free-living invertebrates of Eurasian Arctic seas and adjacent deep waters, Vol. 1. Rotifera, Pycnogonida, Cirripedia, Leptostraca, Mysidacea, Hyperiidea, Caprellidea, Euphausiasea, Dendrobranchiata, Pleocyemata, Anomura, and Brachyura. Alaska Sea Grant, University of Alaska Fairbanks, 2009.
11. Kluge GA. Bryozoans of the Northern seas of the USSR. Moskow–Leningrad: AN SSSR; 1962 (In Russian).
12. Dilman AB. Deep-sea fauna of European seas: An annotated species check-list of benthic invertebrates living deeper than 2000 m in the seas bordering Europe. Asteroidea. Invertebrate Zool. 2014; 11(1): 25–42.
13. Djakonov A.M. Sea stars (Asteroids) of the USSR Seas. Keys to the Fauna of the USSR, 34. Zoological Institute of the Academy of Sciences of the USSR. In Strelkov AA, editor. 1950 (in Russian).
14. Vedenin A N. Budaeva N, Mokievsky V, Pantke C, Soltwedel T, Gebruk A. Spatial distribution patterns in macrobenthos along a latitudinal transect at the deep-sea observatory HAUSGARTEN. Deep Sea Res I. 2016; 114: 90–98.
